# The influence of human papillomavirus type and HIV status on the lymphomononuclear cell profile in patients with cervical intraepithelial lesions of different severity

**DOI:** 10.1186/1750-9378-4-11

**Published:** 2009-08-18

**Authors:** Maria Alice G Gonçalves, Edson G Soares, Eduardo A Donadi

**Affiliations:** 1 Division of Clinical Immunology, University of São Paulo, Ribeirão Preto, São Paulo, Brazil; 2 Department of Pathology, University of São Paulo, Ribeirão Preto, São Paulo, Brazil

## Abstract

**Background:**

Immunological alterations are implicated in the increased prevalence of high-grade squamous intraepithelial lesions (HG-SIL) and persistent human papillomavirus (HPV) infection. This study evaluated the expression of CD4, CD8, CD25 (IL-2Rα) and CD28 antigens from SIL biopsies, stratified by HIV status and HPV-type. Biopsies specimens from 82 (35 HIV^+^) women with a normal cervix, low-grade (LG-SIL) or high-grade lesions (HG-SIL) were studied. CD molecule expression was evaluated by immunohistochemistry and HPV detection/typing performed using PCR techniques.

**Results:**

CD4 stromal staining was increased in patients with HPV18. Women with HPV16 infection showed decreased: a) CD8 and CD25 stromal staining, b) CD25 staining in LG-SIL epithelium and in HG-SIL stroma. In HIV^- ^women samples, CD28 epithelial staining and CD8 stromal staining surrounding metaplastic epithelium were less intense and even absent, as compared to HIV^+ ^women. Both epithelial and stromal CD8 staining was more intense in the HG-SIL/HIV^+ ^group than in the HG-SIL/HIV^- ^group. Positive correlations were observed between CD4/CD25, CD4/CD28 and CD25/CD28 in the stroma and CD25/CD28 in the epithelium.

**Conclusion:**

HIV status and HPV-type may influence the lymphomononuclear cell profile present in the spectrum of cervical lesions. The knowledge of the infiltrating cell profile in cervical tumours may help the development of specific anti-tumoural strategies.

## Introduction

Human Immunodeficiency Virus (HIV)-infected women have a high prevalence of persistent human papilllomavirus (HPV) co-infection and also of squamous intraepithelial lesions (SIL) [1]. The progression from HIV infection status to acquired immune deficiency syndrome is marked by a decreasing CD4 count and increasing HIV viral load, both of which have been associated with the HPV persistence and SIL severity [1,2].

HPV may evade immune surveillance by shifting Th cell polarization, down-regulating the expression of Major Histocompatibility Complex (MHC) class I molecules and reducing the function of intraepithelial antigen-presenting cells, which may cause a lack of Th1 polarization from the beginning of HPV infection. This causes a shift to the production of IL-4, IL-6 and/or the immunosuppressive cytokine IL-10 [3,4]. Similarly, the progression from HIV infection to AIDS has been associated with the polarization to the Th2 cytokine profile in cervicovaginal secretion[5] which might contribute to the persistence of HPV infection [6].

In the general population, tumour infiltrating lymphocytes in cervical cancer have been associated with a depressed function of cytotoxic T cells [7,8]; a down-regulation of CD25 (α chain of the IL-2 receptor-IL-2Rα) activated cells [9] and a decreased proportion of CD4^+ ^T cells with a reversed CD4/CD8 ratio [10]. In HIV-infected women, little information is available regarding the infiltrating T cell phenotypes; however, deregulation of peripheral blood CD4/CD8 T cell function is progressively impaired [11] with hyporesponsiveness of T CD8 cells to activation by the co-stimulatory CD28 and CD40L molecules [12]. To date it is not clear whether these defects in HIV-infected women also occur in the cervicovaginal milieu and whether these might be implicated in the increased prevalence of HG-SIL.

The interaction of the T-cell receptor with the MHC molecule is considered to be the first signal for T cell activation. CD4 and CD8 antigens, observed on the surface of T cells, are co-receptors that bind to non-polymorphic regions of MHC class II and class I molecules, respectively and transduce signals that initiate T cell activation. The second signal for T cells stimulation is provided by the interaction of co-stimulator molecules, including CD28 antigen on T cells and CD80/CD86 on the surface of activated antigen presenting cells. Activation of T cells by antigens and co-stimulators stimulate the production and secretion of IL-2, which in turn induces the production of the IL-2 receptor (IL-2R). The IL-2R is formed by three non-covalently associated protein called a (IL-2Ra), b (IL-2b) and g (IL-2g) chains. The activation of T cells by antigens, co-stimulators and IL-2 leads the expression of IL-2a (CD25) [13]. To determine the possible associations between CD4, CD8, CD25 and CD28 antigens expression in several stages of cervical cancer development, we assessed the expression of these molecules in cervical biopsies stratified according to the severity of the lesion and HPV type detected (HPV16 and 18).

## Materials and methods

### Sample selection

The study was conducted retrospectively on 82 (47 HIV-negative and 35 HIV-positive), non-pregnant, non-lactating, premenopausal women, which were recruited sequentially according to their arrival at the outpatient clinic. They were colposcopically and cytologically screened, selected for cause, i.e., the presence or absence of HPV and cervical intraepithelial lesions. Median age was 36 years (range 19 – 50) for HIV-negative women and 29 years (range 19–47) for HIV-positive women. These women were enrolled at several Gynaecological Reference Services in the State of São Paulo, Brazil, from 1996 to 2001 and the study protocol was approved by the institutional Ethics Committee on human experimentation.

Colposcopies were performed for routine in all women and when indicated, the same physician performed colposcopically directed cervical biopsies or conization loop excision. Cervical fragments were fixed with formalin and then slides were stained with haematoxylin-eosin (Sigma, St. Louis, MO, USA) and histologically evaluated by two experienced histopathologists, who also provided scoring representing the sum of intensity and distribution of staining in fields, in a double-blinded protocol. Thin 5 μm sections were cut, placed on organosilane-pretreated slides and submitted to immunohistochemical assays. An additional 10 μm section was used for DNA extraction and HPV typing.

Cervical biopsies were selected from HIV-negative or HIV-positive women and they were stratified into 3 groups: normal (no evidence of SIL or HPV DNA), low-grade SIL (cervical intraepithelial neoplasia I) and high-grade SIL (cervical intraepithelial neoplasia II-III and *in situ *carcinoma).

### Immunohistochemistry

Serial sections (3–4 μm) obtained from paraffin-embedded blocks, cut and mounted on APTS (3-aminopropyltriethoxy-silane, Sigma, Saint Louis, USA)-pre-treated glass slides were dried by the method of Alves et al. [14]. To prevent cross-reaction with endogenous peroxidase, the slides were treated with 3% H_2_O_2 _for 20 min and then incubated overnight at room temperature with the following primary antibodies at the following dilutions: CD4 (mouse monoclonal, clone 1F6, IgG1 – NovoCastra, NewCastle upon Tyne, UK) 1:80; CD8 (mouse monoclonal, clone 4B1L, IgG2b – NovoCastra), 1:200; CD25 (mouse monoclonal, clone 4C9, IgG2b – NovoCastra) 1:300; CD28 (goat policlonal, clone N20, SC 1625 – Santa Cruz Biotechnology, Santa Cruz, CA, USA), 1:300. Sections of human tonsils were used as positive controls, and a negative control to detect background staining was performed by omitting the primary antibody. Isotype specificity for tonsil sections was confirmed by comparison of staining with irrelevant antibodies of the same isotype as the primary antibody. A control incubation to detect background staining was performed omitting the primary antibody.

After incubation with the primary antibodies, immunoperoxidase staining was performed using a universal biotinylated secondary antibody mixed with a preformed avidin and biotinylated horseradish peroxidase macro-molecular complex (Novostain super ABC kit-NovoCastra), diluted 1:200 for CD4, CD8, CD25 and a different biotinylated secondary antibody (Vector, BA 5000, anti-goat IgG – Burlingame, CA, USA) diluted 1:200 for CD28. Slides were alternately washed 3 times with PBS and TBST (Tris-buffered saline; 0.05 M Tris, pH 7.4, containing 0.3% Tween 20) and diaminobenzidine (0.5 mg/mL) (Sigma) was used as chromogen. Slides were then sequentially counter-stained with haematoxylin without acid for 30 sec, washed in tap water, stained blue with ammoniacal water for 20 sec and exhaustively rinsed with tap water. Finally, slides were dehydrated and mounted for light microscopy evaluation at 400× magnification using an eyepiece graticule connected to an objective lens, as detailed elsewhere [15].

A minimum of 10 fields (total area equivalent to 0.75 mm^2^) was assessed per case. The expression of CD antigens was evaluated using a previously described semi-quantitative method [16]. A total score representing the sum of intensity and distribution of staining in fields presenting dysplastic epithelium and in the respective subepithelial stroma was assigned to each case. The intensity of cellular staining in epithelium and stroma was scored as: (0) no staining, (1) weak; (2) moderate; and (3) intense. Staining in the epithelium was scored as follows: (0), patchy basal; (1), diffuse basal; (2), diffuse full-thickness; (3), patchy and diffuse full-thickness positivity. Staining distribution in stroma was categorised as: (1), patchy subepithelial; (2) diffuse full-extent; (3) patchy and diffuse full-extent positivity. Only sections showing epithelial and stromal tissue were considered for the analysis.

### HPV identification and typing

HPV DNA obtained from paraffin blocks [17] was amplified by PCR using 12.5 pmoles of dNTP, 25 pmoles of each primer, 1.5 U Taq DNA polymerase (Gibco, USA), 5 μL of 10× enzyme buffer, 20 μg of genomic DNA (50 ng) and distilled deionised H_2_O to complete a total volume of 50 μL. The mixture was processed in a thermocycler apparatus (MJ Research, MA, USA) under the following cycling conditions: 1 cycle at 95°C for 5 min, 30 cycles at 95°C for 30 sec, at 55°C for 30 sec and at 72°C for 1 min and finally 1 cycle at 72°C for 10 min and then at 4°C indefinitely.

Since formalin may degrade DNA, producing DNA fragments of different length, several pairs of primers were used. Primers GP5+ and GP6+ [18], which amplify small DNA fragments and primers MY09 and MY11 [19], which amplify longer DNA fragments, were used for generic HPV amplification. Since HPV16 and HPV18 are the types most frequently associated with cervical neoplasia, 2 sets of specific primers were used to detect the E7 gene, one from HPV16 (HPV16E7.667 and HPV16E7.774 primers) and another one from HPV18 (HPV18E7.696 and HPV18E7.799 primers) [20]. All DNAs were amplified with these 4 sets of primers together with a set of primers for a housekeeping gene (globin) [21] as an internal control of amplification. Amplified DNA was applied to a 10% polyacrylamide gel, electrophoresed at 200 volts for 1.45 h and stained with AgNO3 by the method of Sanguinetti et al [22]. It is important to note that the absence of HPV16 or HPV18 did not exclude the presence of HPV types other than 16 or 18, which could be identified if specific primers for them were used. Only unambiguous amplifications were considered as positive. Lack of amplification with these primers or ambiguous amplifications after several repetitions were assigned as other HPV types.

### Statistical Analysis

According to the distribution of the variables and the number of groups compared, statistical analysis was performed using the unpaired t, Mann-Whitney and Kruskall-Wallis tests. The Spearman test (r) was used to calculate correlations. *P *values were two-sided and the level of significance was set at ≤ 0.05. All data were analysed using the Instat Mac 2.01 software (GraphPad software, CA, USA).

## Results

### Patient data

Among the 47 HIV-negative women, 4 exhibited no cervical lesions and 43 presented SIL (13 LG-SIL and 30 HG-SIL). Among 35 women presenting HIV-infection, 5 exhibited no cervical lesions and 30 women presented SIL (21 LG-SIL and 9 HG-SIL). According to CD4 counts, 9 HIV-positive women presented CD4 counts > 500 cells/μL and 26 of them CD4 counts < 500 cells/μL. The median peripheral CD4 T-cell count of the group presenting with both SIL and HIV infection was 360 cells/μL. Patient data stratified by lesion severity (LG-SIL, HG-SIL), HIV status and HPV type are shown in Table 1.

**Table 1:** Data stratified by histopathological diagnosis, HIV status and HPV types.

	**HPV-** **n(%)**			**HPV16** **n(%)**		**HPV18** **n(%)**		**HPV16/18** **n(%)**		**HPVX** **n(%)**	
	
	**Normal**	**LG-SIL**	**HG-SIL**	**LG-SIL**	**HG-SIL**	**LG-SIL**	**HG-SIL**	**LG-SIL**	**HG-SIL**	**LG-SIL**	**HG-SIL**
**HIV-negative (n = 47)**	4 (8.5)	1(2.1)	3(6.4)	5(10.7)	10(21.3)	2(4.3)	6(12.8)	2(4.3)	6(12.8)	3(6.4)	5(10.7)

**HIV-positive (n = 35)**	5 (14.3)	-	-	4(11.4)	1(2.9)	-	2(5.7)	1(2.9)	1(2.9)	16(45.7)	5(14.3)

**Total (n = 82)**	**9(11.0)**	**1(1.2)**	**3(3.7)**	**9(11.0)**	**11(13.4)**	**2(2.4)**	**8(9.8)**	**3(3.7)**	**7(8.6)**	**19(23.2)**	**10(12.2)**

HPV = Human papillomavirus; HIV = Human Immunodeficiency Virus; HPV *X *= Unidentified HPV types; LG-SIL = Low-grade Squamous Intraepithelial Lesion; HG-SIL = High-grade Squamous Intraepithelial Lesion.

### CD4 and CD8 staining

**Figure 1:**
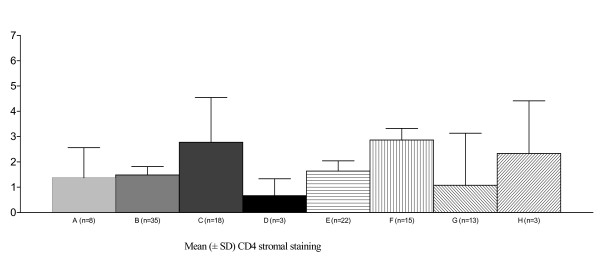
**Increased immunohistochemical expression of CD4 stromal staining of cervical specimens (mean ± SD) in the presence of HPV18 infection (Kruskal-Wallis = 0.02)**. A = HPV-negative; B = HPV18-negative; C = HPV18-positive; D = both HIV and HPV are negative; E = both HIV and HPV18 are negative; F = HIV-negative, HPV18-positive; G = HIV-positive, HPV18-negative; H = both HIV and HPV18 are positive. HPV = Human papillomavirus; HIV = Human Immunodeficiency Virus.

**Figure 2:**
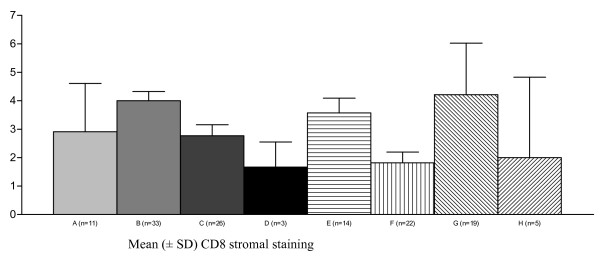
**Reduced immunohistochemical expression of CD8 stromal staining of cervical specimens (mean ± SD) in the presence of HPV16 infection (one way ANOVA test = 0.004)**. A = HPV-negative; B = HPV16-negative; C = HPV16-positive; D = both HIV and HPV are negative; E = both HIV and HPV16 are negative; F = HIV-negative, HPV16-positive; G = HIV-positive, HPV16-negative; H = both HIV and HPV16 are positive. HPV = Human papillomavirus; HIV = Human Immunodeficiency Virus.

**Figure 3:**
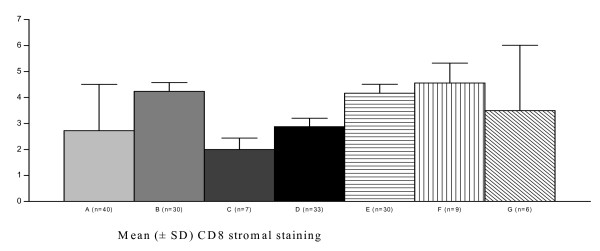
**Increasing immunohistochemical expression of CD8 stromal staining of cervical specimens (mean ± SD) in the presence of HIV-infection (one way ANOVA test = 0.0005)**. A = HIV-negative; B = HIV-positive; C = both HIV and SIL are negative; D = HIV-negative, SIL-positive; E = HIV-positive, SIL-negative; F = both HIV and SIL are positive; G = all HIV, SIL and HPV16 are positive. HPV = Human papillomavirus; HIV = Human Immunodeficiency Virus; SIL = Squamous Intraepithelial Lesion.

CD4 and CD8 staining was observed in cells presenting the morphology of lymphomononuclear cells. CD4 staining was observed throughout the squamous epithelium, and remarkably more intense in the stroma under the transformation zone and surrounding endocervical glands, especially near inflammatory areas. CD4 stromal staining was increased, particularly in specimens presenting HPV18, when compared with those without HPV18 infection. However, in the HIV-positive/HPV18 group, CD4 stromal staining showed to be decreased, when compared with those in the HIV-negative/HPV18 group (*P *= 0.02) (Fig 1). Few koilocytotic cells presented CD8 staining. A significant decreasing of CD8 stromal staining was observed when specimens with HPV16 infection alone were compared with specimens without HPV16. However, CD8 stromal staining seemed to be increased in specimens with both HIV and HPV16 infections, when compared with specimens from patients HIV-negative/HPV16 (*P *= 0.004) (Fig 2). CD8 stromal staining was more frequently localised surrounding the metaplastic epithelium and more intensively in specimens from HIV-positive group, compared to the HIV-negative group (*P *= 0.001). In the HIV-positive/SIL group, clusters of lymphomononuclear cells were observed around vessels and adjacent to the basement membrane. HIV-positive/SIL group exhibited a more intense CD8 stromal staining than observed in the HIV-negative/SIL group (*P *= 0.0005) (Fig 3).

### CD25 (IL-2Rα) and CD28 staining

**Figure 4:**
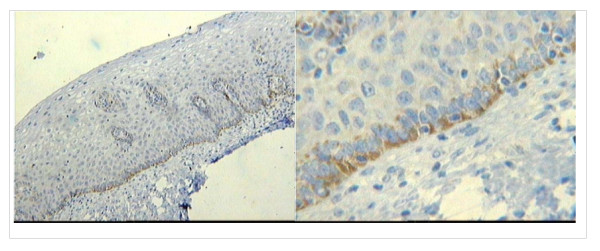
**CD25 staining in a basal and immature cervical squamous epithelium**.

**Figure 5:**
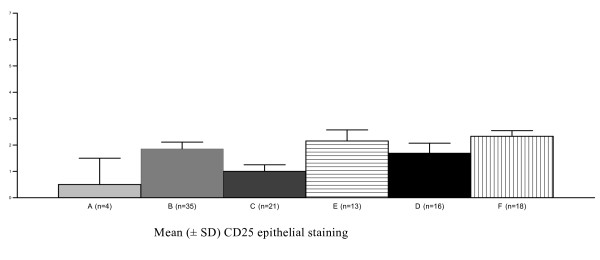
**Reduced immunohistochemical expression of CD25 epithelial staining of cervical specimens (mean ± SD) in the absence of HPV16 infection (Kruskal-Wallis = 0.03)**. A = all HIV, HPV and SIL are negative; B = HPV16 total; C = HPV16-negative, LG-SIL-positive; D = both HPV16 and LG-SIL are positive; E = HPV16-negative, HG-SIL-positive; F = both HPV16 and HG-SIL are positive. HPV = Human papillomavirus; HIV = Human Immunodeficiency Virus; SIL = Squamous Intraepithelial Lesion; LG-SIL = Low-grade Squamous Intraepithelial Lesion; HG-SIL = High-grade Squamous Intraepithelial Lesion.

**Figure 6:**
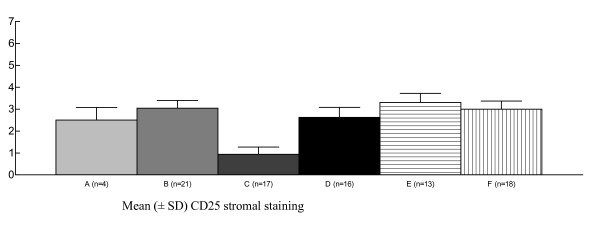
**Reduced immunohistochemical expression of CD25 stromal staining of cervical specimens (mean ± SD) in the absence of HPV16 infection (Kruskal-Wallis = 0.002)**. A = all HIV, HPV and SIL are negative; B = HPV16 total; C = HPV16-negative, LG-SIL-positive; D = HPV16-negative, HG-SIL-positive; E = both HPV16 and LG-SIL are positive; F = both HPV16 and HG-SIL are positive. HPV = Human papillomavirus; HIV = Human Immunodeficiency Virus; SIL = Squamous Intraepithelial Lesion; LG-SIL = Low-grade Squamous Intraepithelial Lesion; HG-SIL = High-grade Squamous Intraepithelial Lesion.

In most specimens, CD25 staining was observed in basal and immature epithelium, independent of HIV status (Fig 4). However, CD25 epithelial staining varied according to both HPV status and lesion severity, being more frequent in the HPV16-positive/HG-SIL group than in the HPV16-negative/LG-SIL group (*P *= 0.03) (Fig 5). CD25 stromal staining was primarily observed in lymphomononuclear cells. More intense CD25 stromal staining was observed in HPV16-negative/HG-SIL group than in the HPV16-negative/LG-SIL group (*P *= 0.002) (Fig 6). Both epithelial and stromal CD25 staining (respectively, *P *= 0.02 and 0.005) were associated with HPV type infection (HPV16, HPV16/18 and other HPV types), when compared to HPV-negative group. No significant difference was found in epithelial or stromal CD25 staining in patients presenting or not HG-SIL, regardless of HIV status.

CD28 stromal staining was observed in lymphomononuclear cells, particularly under the epithelial lesion and under the transformation zone, sometimes as patchy agglomerates, resembling germinative centres. CD28 epithelial staining was less frequently observed in HIV-negative patients than in HIV-positive patients (*P *< 0.0001), irrespective of the HPV type infection or the SIL severity (Data not shown).

Positive correlations were observed between CD4 and CD25 in stroma (r = 0.32, p = 0.01), CD4 and CD28 in stroma (r = 0.33, p = 0.03) and between CD25 and CD28 both in epithelium (r = 0.31, p = 0.03) and in stroma (r = 0.40, p = 0.004).

## Discussion

A cellular infiltration composed essentially of CD4 and macrophages is frequently observed in condyloma under spontaneous regression [23], and a decrease in the number of Langerhans cells in the transformation zone has been associated with a reduced *in vitro *T cell proliferation and IL-2 production [24]. With the progression to HG-SIL, the number of immature Langerhans cells increase with a consequent deficient function [24,25]. In this study, we evaluated the intensity and distribution of CD4, CD8, CD25 and CD28 molecules in cervical specimens with SIL, stratified according to HIV status and HPV type.

In the present study, specimens harbouring HPV18 showed a decreased CD4 stromal staining and HPV16 infection was associated with an increased CD8 stromal staining, in HIV-infected women. Since CD8 cells are recruited preferentially to cervical lesions with progression to invasion [26], and in regressing CIN1 lesions, CD4+ cells predominated within the stroma with highest CD4/CD8 ratio compared with progressive CIN1 [27-29], these findings may explain the progression or regression of the HPV infection towards carcinogenesis, particullarly among HIV-infected women.

HPV16 is considered to be the major etiologic agent of squamous cervical carcinoma and keratinocytes (non-professional antigen presenting cells) are reported to directly present viral antigens to cytotoxic T lymphocytes by cross priming [1], whereas HPV18 is more frequently associated with adenocarcinoma. The mechanisms of viral peptide presentation are less studied than those reported for squamous carcinoma. Increased HPV16 E7-specific T helper cell responses are associated with persistent HPV16 infection, HG-SIL and invasive cervical carcinoma [30]. It is accepted that HPV infects keratinocytes and interferes with the local cytokine expression [31-33], but it is still unknown how HPV together with HIV influences local T cell function.

In this study, CD8 staining increased in HIV infected group and further increased with concomitant SIL. In addition, both the epithelium and stroma of the HIV^+^/SIL group presented an increased expression of CD8 compared to the HIV^-^/SIL group. These findings indicate a continuous stimulation by persistent HPV infection, in agreement with other authors [27,28]. Because of the proximity of the columnar epithelium to the stroma and the lack of keratinocytes in the endocervix, the findings of this study suggest that stromal lymphomononuclear CD4 cells (T CD4 and macrophages) may cope better than CD8 in HPV18 infection. In other words, macrophages may be the major APC in stroma and CD4 cells (T lymphocytes and macrophages) may be involved in immunity against HPV18.

Several studies have shown that peripheral blood lymphocytes produce IL-2[34,35] and generate cytotoxic activity [36] after specific HPV16 E7 protein stimulation. De Gruijl et al. [37] reported an increased IL-2 production by peripheral blood CD4^+ ^T cells, associated with persistent HPV infection and progression of a premalignant lesion and a higher production of Th2 cytokines related to cervical malignancy. In our study, HPV16 associated with decreased stromal CD8 and CD25 expression and increased CD25 staining was observed in both epithelium and stroma with HG-SIL. The expression of IL-2R on keratinocytes and the IL-2 secretion has been reported in different phases of cancer cell development [38,39]. Binding of IL-2 to IL-2R on tumour cells may down-regulate surface expression of IL-2R, the intercellular adhesion molecule-I (ICAM-I) and the MHC class I antigens and may inhibit the *in vitro *growth of tumour cells by arresting these cells in the G0/G1 cell cycle [40]. Although intralesional IL-2 levels were not measured in the present study, the increased expression of CD25 on epithelial cells suggests that IL-2 may control tumour progression.

In this study, irrespective of HPV type and HIV status, significant positive correlations were observed between CD4/CD28 and CD4/CD25 in stroma and between CD25/CD28 in stroma and epithelium of SIL specimens, indicating that CD4 cells are activated, i.e., expressing co-stimulatory molecule (CD28) and responding to IL-2 by expressing CD25. Since IL-2 reportedly induces lymphocyte proliferation/apoptosis depending on IL-2 production and the persistence of the immune response [13], one may hypothesise that lesions able to regress contain more activated CD4 cells, CD28 and CD25 [2]. In contrast, in malignant lesions, tumour-infiltrating lymphocytes are reported to be functionally inhibited, losing their ability of clonal proliferation due to the depression of CD25 [9]. Since less than 2% of CD4/CD8 cells expressing CD25 molecule is observed in the normal endocervix [41] and since CD4/CD25 cells are recognised as regulatory T cells [13], the role of these cells in SIL development should be further evaluated performing longitudinal studies double staining CD4 and CD25.

In conclusion, our results indicate that HPV type and HIV status may influence lymphomononuclear cell marker staining in cervical lesions. A particular immune response may be triggered depending on the balance of these variables, culminating in the progression to cancer. Knowledge of the lymphomonuclear cell profile and the tumour immunogen may help understanding HPV evasion mechanisms and enable new proposals of anti-tumoral treatment, especially therapeutic vaccines against cervical lesions.

## Competing interests

The authors declare that they have no competing interests.

## Authors' contributions

MAG Gonçalves designed and coordinated the work, the results analyses and manuscript writing. EA Donadi participated in the design of the study and in the immunological evaluation and data interpretation, and EG Soares was responsible for slides histopathological interprestion. All authors read and approved the final manuscript.
